# Latitudinal resource gradient shapes multivariate defense strategies in a long‐lived shrub

**DOI:** 10.1002/ecy.3830

**Published:** 2022-09-29

**Authors:** Jordan R. Croy, Jessica D. Pratt, Kailen A. Mooney

**Affiliations:** ^1^ Department of Ecology and Evolutionary Biology University of California Irvine California USA; ^2^ Department of Entomology University of Georgia Athens Georgia USA

**Keywords:** environmental gradients, local adaptation, plant defense, resource availability hypothesis, syndromes, trade‐offs

## Abstract

Plant defense against herbivores is multidimensional, and investment into different defense traits is intertwined due to genetic, physiological, and ecological costs. This relationship is expected to generate a trade‐off between direct defense and tolerance that is underlain by resource availability, with increasing resources being associated with increased investment in tolerance and decreased investment in direct resistance. We tested these predictions across populations of the shrub *Artemisia californica* by growing plants sourced from a latitudinal aridity gradient within common gardens located at the southern (xeric) and northern (mesic) portions of its distribution. We measured plant growth rate, resistance via a damage survey, and tolerance to herbivory by experimentally simulating vertebrate herbivory. Plants from more northern (vs. southern) environments were less resistant (received higher percent damage by vertebrate herbivores) and tended to be more tolerant (marginally significant) with respect to change in biomass measured 12 months after simulated vertebrate herbivory. Also, putative growth and defense traits paralleled patterns of resistance and tolerance, such that leaves from northern populations contained lower concentrations of terpenes and increased N, specific leaf area, and % water. Last, plant growth rate did not demonstrate clear clinal patterns, as northern populations (vs. southern populations) grew more slowly in the southern (xeric) garden, but there was no clinal relationship detected in the northern (mesic) garden. Overall, our findings support the prediction of lower resistance and higher tolerance in plant populations adapted to more resource‐rich, mesic environments, but this trade‐off was not associated with concomitant trade‐offs in growth rate. These findings ultimately suggest that plant adaptation to resource availability and herbivory can shape intraspecific variation in multivariate plant defenses.

## INTRODUCTION

Plants have evolved an array of defensive strategies to cope with damage by herbivores. Plant defenses can be classified into two broad strategies, resistance and tolerance, which are the plant's ability to reduce herbivory and mitigate the fitness costs of herbivory, respectively (Strauss & Agrawal, 1999). However, the costs of herbivore defense can constrain plant investment in each strategy and generate trade‐offs between tolerance and resistance (Agrawal, 2020; Mooney et al., 2010; Züst & Agrawal, 2017). In contrast, positive correlations among plant defense traits can arise when defense traits act synergistically, or when there is parallel selection by different selective agents on separate defense traits (Agrawal, 2020; Agrawal & Fishbein, 2006; Hahn & Maron, 2016). Identifying the processes that shape tolerance and resistance can provide a framework for predicting plant defenses and their downstream ecological and evolutionary consequences (Agrawal, 2020; Fornoni, 2011; Nunez‐Farfan et al., 2007; Tiffin, 2000).

Resource availability has been proposed to underly patterns of genetic variation in plant tolerance and resistance through two contrasting dynamics. One long‐standing hypothesis asserts that resource availability acts strictly through a bottom‐up dynamic, determining genetic‐based variation in growth rate, and faster growth rates are assumed to confer greater tolerance to herbivore damage. This increased tolerance in turn reduces the selection for plant resistance to herbivory to generate a tolerance–resistance trade‐off (i.e., resource availability hypothesis [RAH]; Coley et al., 1985; Endara & Coley, 2011; Fine et al., 2004). Alternatively, bottom‐up effects of resource availability on plant growth and tolerance may be complicated by a feedback effect in which increased resource availability results in greater herbivore pressure that exceeds plant tolerance capacity. This increased herbivory in turn selects for plant resistance to generate a positive relationship between tolerance and resistance (Hahn & Maron, 2016; López‐Goldar et al., 2020; Moreira et al., 2017). However, given that tolerance and resistance have been shown to share the same molecular pathway, molecular constraints on a tolerance–resistance trade‐off can also lead to positive covariation between the two defense strategies along a resource gradient (Mesa et al., 2017; Scholes et al., 2017; Scholes & Paige, 2015). The bottom‐up dynamic has largely been applied to the study of interspecific variation in plant growth and defense (Coley et al., 1985; Endara & Coley, 2011), whereas the feedback dynamic has been proposed to underlie patterns of intraspecific variation (Hahn & Maron, 2016). However, fewer studies have quantified plant growth‐defense patterns within species and there is support for both the bottom‐up (Hahn et al., 2021; Pratt et al., 2017; Pratt & Mooney, 2013) and feedback dynamic (Hahn et al., 2018; López‐Goldar et al., 2020). Therefore, more studies are needed to resolve under which conditions bottom‐up versus feedback dynamics shape plant growth‐defense patterns.

Essential to the RAH is the prediction that tolerance is greater for plants from high‐ versus low‐resource environments, although tolerance is rarely measured. Instead, growth rates are often used as a proxy for tolerance with the assumption that fast‐growing plants can more easily replace tissue lost to herbivores. Although tolerance and growth may correlate positively (Gianoli & Salgado‐Luarte, 2017), fast relative growth rates may come at the cost of regrowth capacity (Rose et al., 2009; Strauss & Agrawal, 1999), leading to trade‐offs between relative growth rate and tolerance (Croy et al., 2020; Scholes et al., 2017), or plants may respond by increasing overall vigor (Stowe et al., 2000; Turley et al., 2013). Moreover, we know little about how adaptation to resource availability might influence tolerance to herbivory. Previous studies have documented genetic‐based variation in tolerance to herbivory along latitudinal gradients (Croy et al., 2020; Lehndal & Ågren, 2015; but see Sakata et al., 2017; Więski & Pennings, 2014), suggesting that plant adaptation to the abiotic and biotic environment influences tolerance.

The objective of this study was to test whether resource availability shapes intraspecific variation in plant growth and defense through a bottom‐up versus a feedback dynamic. For a latitudinal gradient in resource availability characterized by a northward decrease in aridity, we tested whether plant resistance, tolerance, and growth would increase with plant latitude of origin in accordance with the feedback dynamic. To this end, we used a series of common gardens comprised of 21 populations of *A. californica* sourced along a 5° latitudinal cline containing a northward increase in mean annual precipitation and soil conditions associated with greater resource availability. Within a single common garden located at the southern end of the species' distribution, we measured a series of growth and defense traits (e.g., % nitrogen, terpene concentration, specific leaf area [SLA]) and surveyed plants for damage to assess resistance, and then experimentally manipulated herbivory to quantify tolerance. Within two gardens located at the southern and northern ends of the species' distribution, we measured plant growth rate. By simultaneously quantifying plant resistance, tolerance, and growth, we sought to assess plant growth‐defense relationships with the explicit consideration of plant tolerance to herbivory and resource availability.

## METHODS

*Artemisia californica* (Less. Asteraceae) is a dominant, long‐lived shrub of California's biodiverse and threatened coastal sage scrub ecosystem (Myers et al., 2000). This shrub spans a 1000 km distribution that encompasses a five‐fold precipitation gradient from Northern Baja, Mexico (average annual precipitation: 20 cm) to Mendocino County, California (average annual precipitation: 103 cm). Recent studies have documented genetic‐based trait variation across populations of *A. californica* that is suggestive of local adaptation (Pratt & Mooney, 2013). Ecotypes vary in their ability to tolerate extreme drought (Croy et al., 2021), and these ecotypic differences in turn influence the abundance and community composition of arthropods (Croy et al., 2021; Pratt et al., 2017) that are both a key component of biodiversity and support several endemic and endangered vertebrates that drive regional conservation efforts (Bowler, 2000).

Vertebrate herbivore communities in coastal sage scrub ecosystems are diverse, consisting of squirrels (*Sciurus niger* and *Otospermophilus beecheyi*), rabbits (*Sylvilagus audubonii*), and woodrats and mice (*Nassella lepida*, *Nyctinomops macrotis*, *Peromyscus eremicus*, and *Cantharellus californicus*) (Quon et al., 2019). Some prefer young grasses or forbs over fresh shoots of *A. californica* during the growing season, but shift their feeding onto shrubs during the summer and fall when shrubs are particularly vulnerable (Genin & Badan‐Dangon, 1991; Genin & Pijoan, 1993). Vertebrate herbivory can substantially reduce shrub performance and alter plant community composition (Callaway & Davis, 1993; Freudenberger et al., 1987). For instance, Quon et al. (2019) found that herbivores reduced the biomass of uncaged *A. californica* seedlings by 57%, which overall highlights the important but understudied role that vertebrate herbivores play in shaping coastal sage scrub dynamics (Litle et al., 2019). Moreover, the chemical composition of *A. californica* has been invoked as a potential limiting factor of vertebrate herbivory (Duke et al., 1987; Halligan, 1975; Jogia et al., 1989).

### Common garden design

This study is based upon the analysis of data from three common gardens, one containing five populations and established in 2009, and two containing 21 populations and established in 2011 (Appendix S1: Table S1). The 2009 garden is located in Newport Beach, CA (33°39′ N) and within the Upper Newport Bay Ecological Preserve. Wild *A. californica* grows within 10 m of the garden perimeter. The site has a mean annual precipitation and temperature (from 1964 to 2014) of 29.9 cm and 17.6°C, respectively (Appendix S1: Table S1). The details regarding common garden construction are reported elsewhere (Croy et al., 2021; Pratt & Mooney, 2013), but the core design is briefly described here. For the common garden established in 2009 (from this point forwards the “2009 garden”), cuttings from five *A. californica* populations were collected along a coastal gradient in spring 2008 and grown within a greenhouse. In December 2009, the common garden was planted into three blocks each containing a pair of plots, one irrigated and the other unirrigated (Pratt et al., 2014, 2017; Pratt & Mooney, 2013). The plants from each source population (sample sizes ranging from 7 to 21 per population) were evenly distributed among plots and randomized within each plot. To minimize nongenetic maternal effects associated with plants cloned from cuttings (Roach & Wulff, 1987), rooted cuttings were grown in the greenhouse and common garden for a total of 24 months before collecting data.

In 2011, we established two identical common gardens at the northern and southern end of the *A. californica*'s distribution (from this point forwards the “2011 gardens”). The southern garden is located directly adjacent to the 2009 garden described above. The northern garden is located within a coastal prairie habitat at the Jenner Headlands Preserve in Jenner, California (38°27′N), and has a mean annual precipitation and temperature (from 1964 to 2014) 107.6 cm and 11.6°C, respectively. In December 2010, we collected seed from 10 *A. californica* plants in each of 21 source populations (Appendix S1: Table S1). Seeds were germinated in early February 2010 in a greenhouse. In February 2011, when the plants reached a canopy volume of ~1000 cm^3^ (≈10 × 10 × 10 cm), they were transplanted to each common garden site with ~10 individuals per population (*N* = 210 plants total), each from a unique seed mother. Plants were randomly assigned to locations within a 14 by 15 m grid, with each plant separated by 1.0 m from its closest neighbor. Plants within each garden were lightly irrigated during their first summer following transplant to increase survival. We conducted our tolerance experiment in the southern 2011 garden, assessed vertebrate herbivory in both the 2009 and 2011 southern common gardens, and measured plant size in each of the 2011 common gardens (Appendix S1: Table S2).

### Latitudinal variation in resource availability and herbivory

We characterized the latitudinal gradient with respect to aridity, soil properties, and vertebrate and invertebrate herbivory. For aridity, we extracted PRISM climate data from 1970 to 2000 (4 km spatial resolution; PRISM Climate Group 2004). These climate variables were then used to calculate potential evapotranspiration (PET) using the Hargreaves equation following the protocol of the Consortium for Spatial Information (CGIARCSI) Global Aridity and PET database. From this, we calculated a unitless Aridity Index (mean annual precipitation/mean annual potential evapotranspiration), which is the inverse of aridity, with low values indicating more arid locations. Full details are provided in Appendix S1: Section S2.

Using the USDA NRCS SSURGO database, we extracted percent sand, silt, and clay because of their relationship to water storage capacity and other indices of resource availability (Appendix S1: Figure S2). Each population occurs within a distinct soil type called a map unit. Each map unit is comprised of various soil components (component units), and the proportion of each component unit varies depending on the map unit. Moreover, each component unit contains unique soil horizon data. Soil properties were specifically extracted from the *chorizons* table within the SSURGO database. The chorizons table contains information on soil attributes at various soil depths, but because the majority of *A. californica* roots are concentrated within the first 50 cm of soil (Goldstein & Suding, 2014), we calculated means weighted by soil depth. Due to the multivariate nature of soil, we used the *prcomp* function in R (R Core Team, 2019) to perform a principal components analysis on percentage sand, silt, and clay (each *z*‐transformed) in order to collapse soil variables into single principal component before using in subsequent analyses.

### Plant growth and defense traits

We measured a suite of leaf‐level traits that are widely recognized to influence plant growth and defense against herbivory (Pérez‐Harguindeguy et al., 2016) to assess how functional traits relate to plant growth and defense strategies. In April 2014, during peak growing season, we collected 30 fully expanded leaves from five plants within each population; 10 leaves were used to assess SLA and percentage water content (%WC), 10 leaves were used to assess plant defensive chemistry (i.e., terpenes), and the remaining 10 leaves for nitrogen analysis. For SLA and PWC, freshly picked leaves were immediately placed on ice and kept cool until they were scanned and weighed (wet weight) later that same day. Leaves were then dried at 60°C for 72 h and weighed again (dry weight). Leaf area (cm^2^) was determined from scanned images using ImageJ software (Rasband, 2008). SLA was calculated as mm^2^ per mg^−1^ dry weight and PWC as (wet weight − dry weight)/wet weight. To assess leaf nitrogen (N) content, leaves were dried at 60°C for 72 h and then ground to a fine powder using a Wig‐L‐bug grinding mill (International Crystal Laboratories, Garfield, NJ). Approximately 1 mg of this homogenized powder was then packed into 5 × 9 mm tins. Elemental analysis (Fisons Instruments 1500) and mass spectrometry (Delta plus XL, Thermo Finnigan, Asheville, NC) was then performed at the UC‐Irvine Stable Isotope Ratio and Mass Spectrometry Facility. Terpene concentrations were assessed using gas chromatography and mass spectrometry (GC–MS), the details of which are described in Pratt et al. (2014) and also included in Appendix S2.

### Vertebrate herbivory

Vertebrate herbivory on *A. californica* is concentrated in late summer and early fall (Litle et al., 2019). Desert cottontails (*S. audubonii*) were one of the dominant herbivores observed at our southern common garden location, and we observed that herbivores often discarded stems into stems piles underneath a shrub canopy. To quantify damage on plants, we visually assessed the proportion of a plant's canopy that was damaged by herbivores in spring of 2010 in the 2009 common garden. In the field, damage on plants was assigned to one of six bins: no damage, <10%, 11%–25%, 26%–50%, 51%–75%, >75%. Prior to analysis, bins were converted to a continuous variable of percent estimated canopy damage by taking the midpoint of each bin. To estimate damage within the 2011 garden, we collected clippings from beneath the shrub canopy that were left behind by herbivores twice in mid to late summer of 2016. We weighed the clippings and summed the mass across the two time points to get a measure of total herbivore damage. Because we swept debris from beneath the plant in early June, the clippings we collected represent the total amount of damage over a three‐month period. The biomass of the clippings was divided by total plant biomass to obtain a percentage of aboveground biomass removed and discarded by herbivores. Although it is not clear why herbivores discard plant tissue, this may not be relevant from a plant's perspective given that tissues is still being removed. Also, we were unable to relate these two measures of vertebrate herbivore damage directly due to only five overlapping populations between the two gardens. However, the parallel relationship of each herbivore damage estimate to population latitude of origin suggests that clippings can serve as a proxy for total herbivory received by *A. californica* plants.

### Simulated folivory treatment and fitness measurements

We implemented a clipping treatment to simulate the effects of heavy folivory. We used artificial herbivory treatment (clipping) to minimize variation in herbivory levels among plants. In May of 2016, we sorted plants within each population into two groups containing approximately equal biomass to standardize the starting conditions for each treatment ($\chi^{2}$ = 1.55, *p* = 0.213). We then randomly assigned each group to either a “clipped” or “unclipped” control. In the spring of 2016, we then removed 50% of the aboveground photosynthetic material with scissors to simulate the median magnitude of feeding damaged observed in the field (J. R. Croy, pers. observs.).

To assess the effects of folivory on plant performance, we measured plant canopy size at the conclusion of the growing season (mid‐May) in 2016 and 2017, and plant survival in 2017. To estimate aboveground dry biomass, we collected reference branches from an *A. californica* shrub outside of our garden plots and estimated the total number of such branches needed to reconstruct our experimental shrubs separately for two reference branches. These reference branches were then dried and weighed in order to estimate shrub dry biomass. We estimated the growth rate of each population using two methods. First, because plants were established at the same time, we treated the biomass estimates from 2018 in the southern and northern garden as one indicator of growth rate. Second, in the southern garden alone, we estimated growth rate of individuals from 2016 to 2017 using a log response ratio, in which growth = ln[biomass_2017_/biomass_2016_] (Hedges et al., 1999). Growth values above, equal to, or below zero indicate plant growth, stasis, and shrinkage, respectively.

### Statistical analysis

The objective of this study was to test for latitudinal clines in (1) abiotic conditions related to resource availability and (2) plant resistance, tolerance, and growth to shed light on how a latitudinal gradient in resource availability and herbivory combine to shape genetically based variation in plant growth‐defense strategies. Clinal variation in resource availability was assessed by regressing source site aridity and soil texture (represented by the first principal component described in *Latitudinal variation in resource availability and herbivory*).

To test the hypothesis that resource availability alters relative investment into tolerance and resistance, we first *z‐*transformed our measures of resistance, tolerance, and growth to standardize their units. Resistance was measured as the inverse of percent clipping damage and tolerance was measured as the log response ratio of plant biomass in 2017 in the clipped versus unclipped treatments (ln[biomass_2017_clipped_/biomass_2017_unclipped_]). We then regressed *z*‐transformed defense values (centered on the mean for resistance and centered on zero for tolerance) against latitude of origin, defense strategy (resistance vs. tolerance), and a latitude by strategy interaction. A significant interaction would indicate that relative investment into resistance versus tolerance depends on a population's latitude of origin. For growth, we *z*‐transformed biomass estimates (after cubed‐root transformation to achieve normality) for both the northern and southern gardens and regressed biomass against latitude of origin, garden, and a latitude by garden interaction to test for clinal variation plant growth rate while accounting for plasticity arising from plants growing in different garden conditions. We then followed up with independent assessments of latitudinal clines in plant defense and growth (i.e., herbivore damage, putative growth, and defense traits). To do this, we constructed a series of linear mixed effects models each containing, at a minimum, latitude of origin as a predictor and plant population as a nested random effect. However, to test for latitudinal variation in plant tolerance, we included a clipping and a clipping by latitude interaction as predictors of plant growth and survival 1 year after damage. Plant growth from 2016 to 2017 was computed via a log response ratio—log$\left(\frac{{\text{biomass}}_{2016}}{{\text{biomass}}_{2017}}\right)$—and plant size in 2016 was included as a covariate to account for the effects of initial plant size. We further modified the survival model with a generalized linear mixed effects model to specify a binomial distribution. For plant growth and survival, a significant interaction between clipping and latitude would indicate latitudinal variation in tolerance. To test for genetic‐based clines in growth rate, we regressed the plant size (estimated in 2018 in the southern and northern 2011 gardens) against latitude of origin, garden location, and their interaction. For estimates of herbivore damage within the 2009 garden, we constructed a linear mixed effects model regressing percent canopy damage against latitude, irrigation treatment, and a latitude by irrigation interaction, while including a plot by population random effect to account for plot‐level variation and nonindependence among individuals within a population, respectively.

We recognize that phenotypes are ultimately expressed at the genotypic level. However, in establishing common gardens, selecting plants for subsampling for leaf trait analyses, and assigning plants to clipping treatments, we consistently randomized our selection of plants, in this case “genotypes.” Therefore, whereas we are not able to precisely estimate genotypic expression of plant growth and defense, we are able to make inferences about how populations of *A. californica* on average grow and defend themselves.

Response variables were ln transformed (cubed‐root transformed for plant biomass) as needed to normalize the distribution of the residuals and normality was assessed via a Shapiro–Wilks test of normality. All analyses were performed in R 3.6.0 (R Core Team, 2019). Linear mixed effects models and sums of squares were constructed and computed using the *lme4* (Bates et al., 2015) and *car* (Fox & Weisberg, 2019) packages in R, respectively.

## RESULTS

### Clinal variation in aridity and soil

**FIGURE 1:**
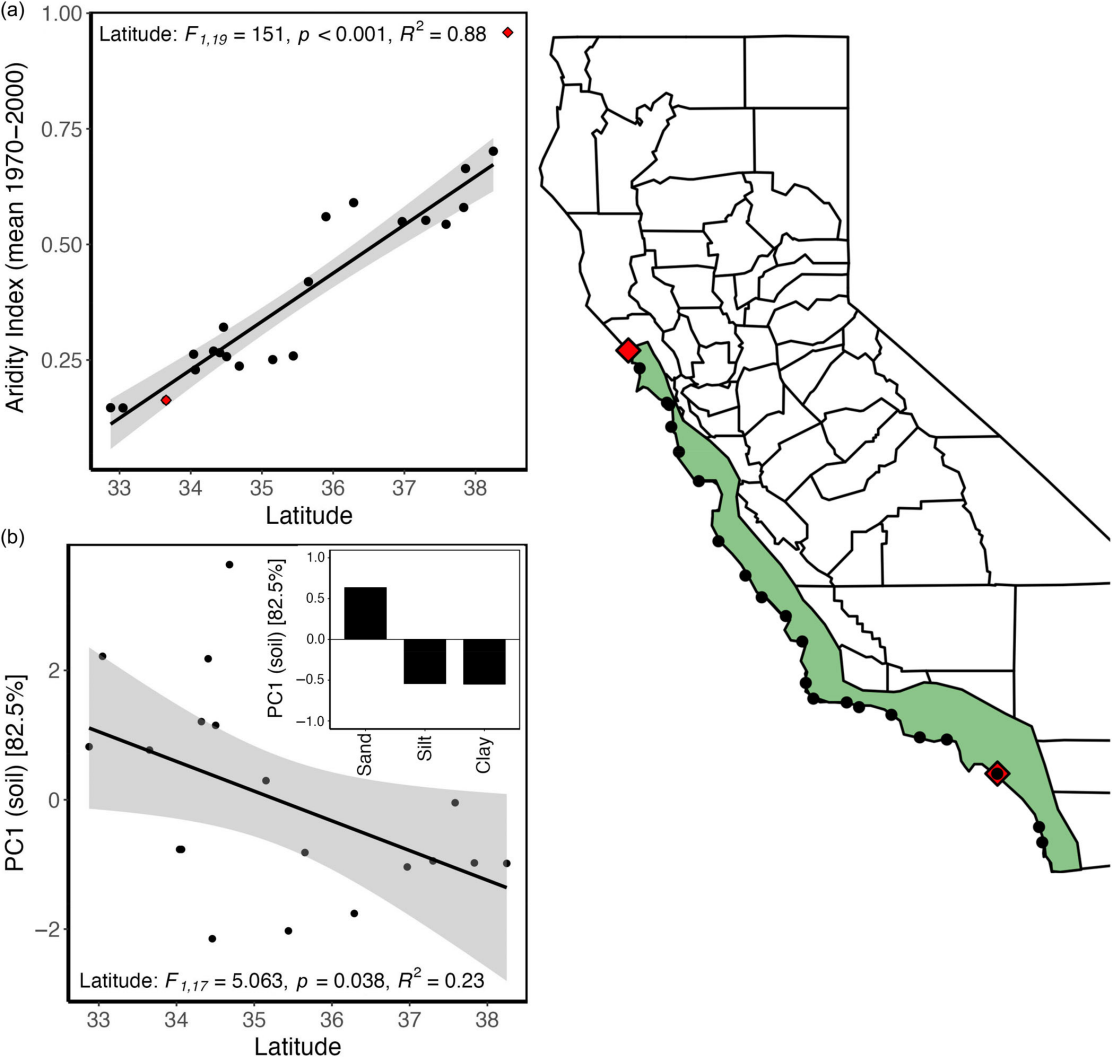
Latitudinal clines in (a) aridity (lower values indicate greater aridity) and (b) soil properties from populations of *Artemisia californica* sourced along California's coast. Soil properties were condensed via principal component, in which the first principal component summarizes 82.5% of multivariation variation in percentage sand, silt, and clay. Loadings for each soil variable onto soil PC1 are displayed within panel (b). Points on map indicate the *A. californica* populations used in this study, as well as the common garden locations (large red diamonds). Green coastal polygon indicates the distribution of *A. californica* in California.

We found significant latitudinal variation in aridity (*F*_1,19_ = 151, *p* < 0.001, *R* ^2^ = 0.88; Figure 1a) and soil properties (*F*_1,17_ = 5.063, *p* = 0.038, *R* ^2^ = 0.23; Figure 1b) across the 20 populations of *A. californica* used in this study. Aridity decreased with latitude, whereas soils became increasingly more clayey, less sandy, and more silty with latitude.

### Clinal variation in defense and growth

**FIGURE 2:**
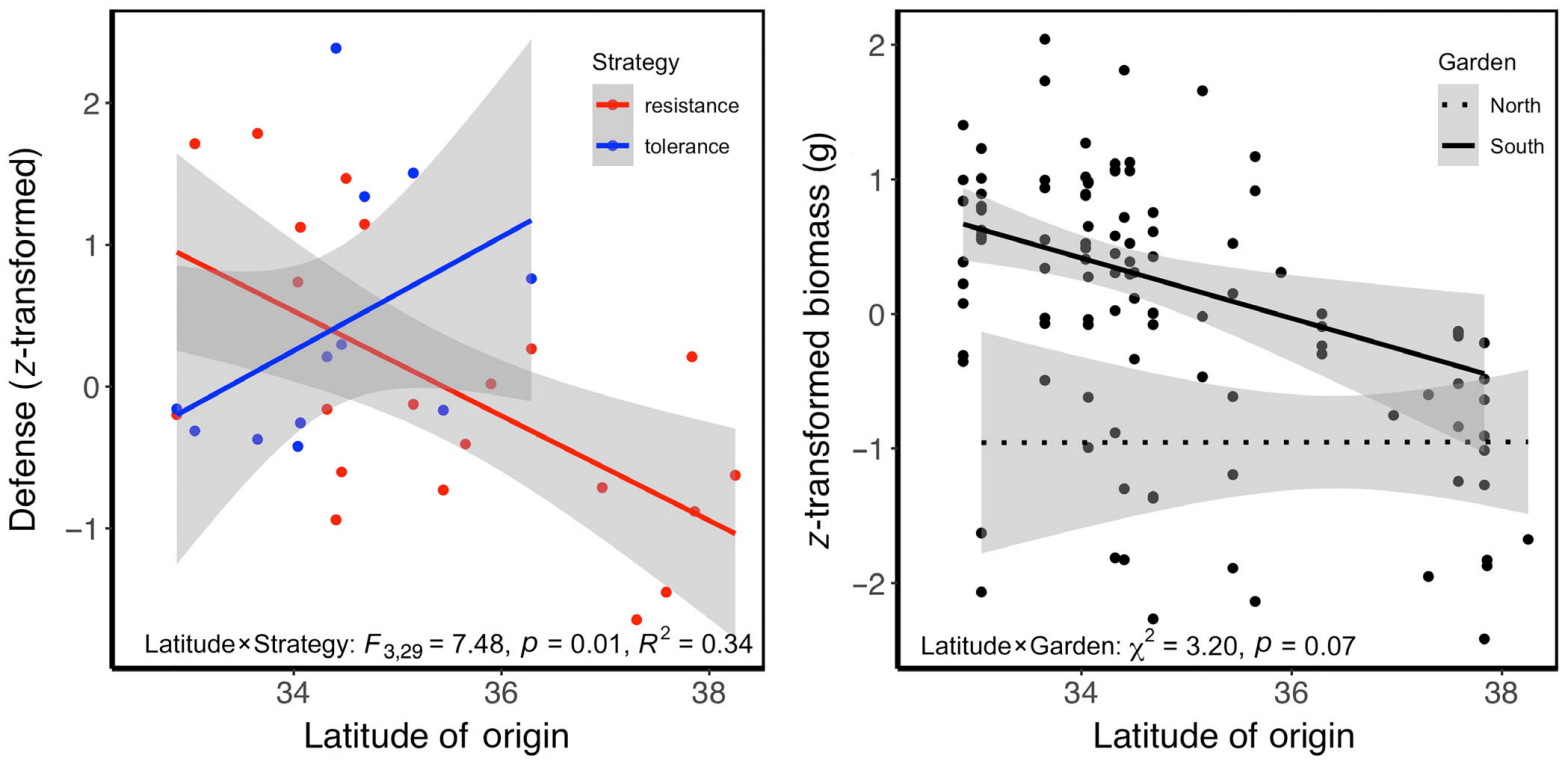
(Left) The relationship between *z‐*transformed defense values for resistance (red) and tolerance (blue) versus population latitude of origin. (Right) *z*‐transformed growth versus population latitude of origin within the northern (dotted) and southern (solid) common garden. Tolerance was calculated as log$\left(\frac{{\text{biomass}}_{2017\_\text{clipped}}}{{\text{biomass}}_{2017\_\text{unclipped}}}\right)$ and resistance as the inverse of percent herbivory estimated via clippings collected beneath *Artemisia californica* canopies. Least squares regression lines plotted with shaded regions indicating 95% CI.

Population latitude of origin and defense strategy interacted to influence defense investment (*F*_3,29_ = 7.480, *p* = 0.010, *R* ^2^ = 0.34; Figure 2). Populations from southern habitats invested relatively more in resistance than tolerance to herbivory, whereas populations from northern habitats invested more in tolerance than resistance (Figure 2). Accounting for plant size, vertebrate herbivory increased with plant latitude of origin ($\chi^{2}$ = 6.492, *p* = 0.011; Appendix S3: Figure S1). From the southern‐ to northern‐most population, percentage herbivory increased from 2.8% to 7.2%. We corroborated this pattern within the five‐population garden, where we found a significant increase in estimated vertebrate damage on plants sourced from south to north ($\chi^{2}$ = 9.102, *p* = 0.003; Appendix S3: Figure S1). In contrast, absolute herbivory, which represents the total amount of tissue removed not accounting for variation in plant size, declined with plant latitude of origin by 52% ($\chi^{2}$ = 6.767, *p* = 0.009; Appendix S3: Figure S2) because northern plants were smaller than southern plants. Due to the wide variation in plant size, the proportion of tissue removed is a better indicator of plant resistance and illustrates the impacts of herbivory from the plant's perspective, whereas absolute herbivory describes where vertebrate herbivores might spend their time foraging (herbivore perspective). Full model results are detailed in Appendix S3: Table S1.

Results were consistent with, but somewhat equivocal for, increased tolerance in plants adapted to resource‐rich environments. The clipping by latitude interaction was marginally significant for plant growth ($\chi^{2}$ = 3.139, *p* = 0.076; Appendix S3: Figure S3) and nonsignificant for survival ($\chi^{2}$ = 2.044, *p* = 0.153; Appendix S3: Figure S3). However, the patterns for both plant growth and survival were consistent with predictions for greater tolerance for plants sourced from high‐resource environments. With respect to the (marginally significant) result for plant growth, plants from southern environments fully compensated for clipping damage, whereas plants from northern environments overcompensated (Appendix S3: Figure S3). With respect to plant survival, plants from southern environments had reduced survival from clipping damage, whereas plants from northern environments maintained survival. Accordingly, in both cases the patterns are suggestive of greater tolerance in plants adapted to the increased resources of mesic environments.

Results for growth did not support the prediction of increased growth rate for plants adapted to resource‐rich mesic environments. Assessed across both the southern and northern gardens, growth rate was significantly affected by latitude ($\chi^{2}$ = 3.882, *p* = 0.049) and garden ($\chi^{2}$ = 23.221, *p* < 0.001) with higher growth from southerly (vs. northerly) in the southern garden. However, the garden by latitude interaction was marginally significant ($\chi^{2}$ = 3.201, *p* = 0.074; Figure 2), suggesting that this latitudinal cline in growth rate depended on garden. Plant growth was associated with latitude of origin in the southern common garden, with plants from more northern environments having lower growth rates ($\chi^{2}$ = 6.954, *p* = 0.008), but there was no association observed in the northern common garden ($\chi^{2}$ = 0.346, *p* = 0.556).

### Clinal variation in leaf growth and defense traits

**FIGURE 3:**
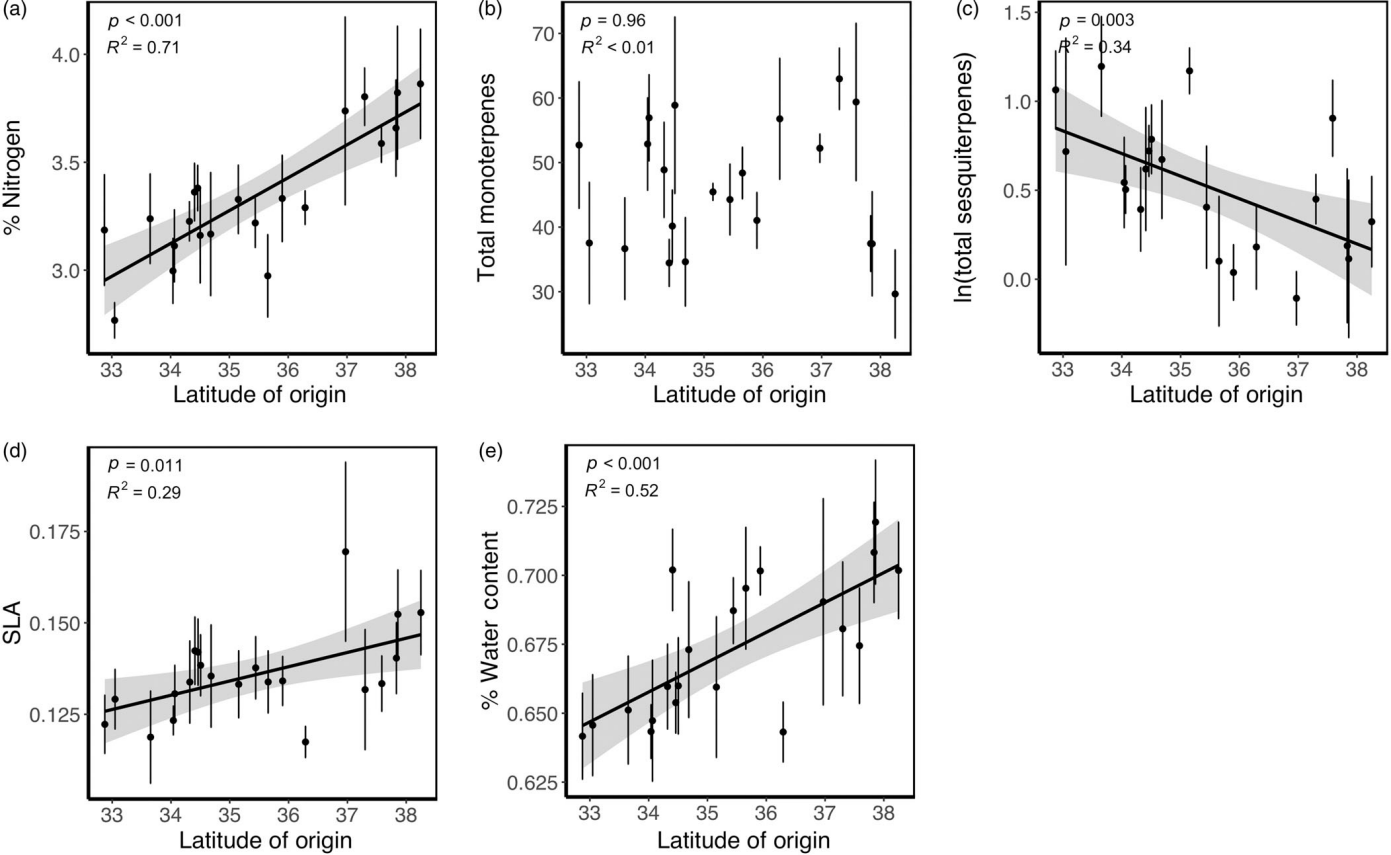
Latitudinal variation in *Artemisia californica* leaf (a) % nitrogen, (b) total monoterpenes, (c) total sesquiterpenes, (d) specific leaf area, and (e) % water content, each putatively associated with plant resistance and tolerance to herbivory. Least squares regression lines plotted with shaded regions indicating 95% CI for significant relationships.

We found significant latitudinal variation in four of the five traits measured. From the southern‐ to northern‐most population, leaf percentage nitrogen, SLA, and %WC increased 28% ($\chi^{2}$ = 32.673, *p* < 0.001; Figure 3a), 17% ($\chi^{2}$ = 6.492, *p* = 0.011; Figure 3d) and 9% ($\chi^{2}$ = 17.372, *p* < 0.001; Figure 3e) with latitude, respectively, whereas total sesquiterpenes decreased 81% ($\chi^{2}$ = 8.528, *p* = 0.003; Figure 3c). Monoterpene concentrations did not vary with latitude ($\chi^{2}$ = 0.003, *p* = 0.959; Figure 3b).

## DISCUSSION

We provide evidence that a latitudinal resource gradient has shaped microevolutionary patterns of plant defense strategies in *A. californica*. We sampled plants from populations adapted to a coastal resource gradient characterized by mesic conditions in the north and arid conditions in the south. Within a common garden, we found that plants from northern populations had leaves associated with higher quality (lower resistance) for herbivores and greater regrowth capacity, including more nitrogen, higher WC, lower sesquiterpene concentration. Correspondingly, these plants received more damaged by vertebrate herbivores, and were marginally more tolerant to a 50% damage treatment than populations sourced from southern environments. With respect to growth rate, results were equivocal, with northern populations being either indistinct or more slow growing than southern populations depending on the common garden setting (northern and southern gardens, respectively). Collectively, these results support some but not all of the theoretical predictions for the distribution of herbivore defense strategies along a resource gradient; populations from high‐resource (northern) environments exhibited lower resistance and higher tolerance (marginally significant) but not faster growth rates compared with populations from low‐resource (southern) environments. Overall, the latitudinal clines in plant defense strategies uncovered here highlight the general importance of environmental gradients in shaping the evolution of plant ecological strategies within species.

Vertebrate herbivores disproportionately damaged northern versus southern plants and intraspecific variation in leaf traits suggests that northern plants are also more palatable to herbivores than southern plants. Plant traits such as terpene concentration, nitrogen content, WC, and SLA (as a surrogate for leaf toughness) have all been linked to herbivore performance (Aharoni et al., 2005; Bleeker et al., 2012; Casotti & Bradley, 1991; Huberty & Denno, 2004; Lucas et al., 2000; Mattson Jr, 1980), and therefore these traits might underly clinal variation in resistance. However, some of these traits (e.g., nitrogen and WC and SLA) have also been evoked at putative growth and tolerance traits (Quijano‐Medina et al., 2019; Tiffin, 2000), complicating interpretations of the adaptive significance of these traits. Interestingly, this latitudinal cline in resistance to vertebrate herbivores parallels a previously documented latitudinal cline in resistance against insect herbivores (Pratt et al., 2017), suggesting that plant local adaptation to a latitudinal coastal aridity gradient has generated a strategy of crossresistance to a diverse set of herbivores. Similar patterns of crossresistance to vertebrate and invertebrate herbivores have also been documented in species of *Eucalyptus* (Andrew et al., 2007) and *Betula* (Rousi et al., 1997). Moreover, the herbivorous insects on *A. californica* are comprised primarily of sap‐feeding insects (Pratt et al., 2017), whereas the vertebrate herbivores are leaf chewers and browsers (Litle et al., 2019). Given that sap‐feeding and leaf‐chewing herbivores have been shown to elicit different phytohormonal pathways (Smith et al., 2009), these results suggest that these responses may nonetheless be correlated and that latitudinal variation in environmental conditions might underly their relative co‐expression.

Latitudinal and elevational clines in plant resistance have been the focus of much empirical investigation, with support for increasing resistance toward the tropics (Baskett & Schemske, 2018; Cronin et al., 2015; Więski & Pennings, 2014) and toward lower elevations (Dostálek et al., 2016; Moreira et al., 2017; Rasmann et al., 2014). These patterns of resistance might arise due to gradients in the strength of herbivory (Coley & Aide, 1991; Johnson & Rasmann, 2011; Schemske et al., 2009). However, we did not find clinal variation in vertebrate herbivore abundance (Appendix S1: Figure S3), and so it is not clear that vertebrate herbivore pressure alone is responsible for driving the observed latitudinal clines in plant resistance; however, we interpret the lack of clinal variation in herbivore pressure with caution, given that the citizen science data collected from *iNaturalist* may be too crude to capture trends in herbivore abundance. In contrast, aridity often varies steeply with latitude and elevation and has been linked to greater plant resistance against herbivory (Anstett et al., 2018; Kergunteuil et al., 2019; Vázquez‐González et al., 2019). Counter to global‐scale patterns of aridity in which temperate regions are more arid than tropical regions, aridity decreases with latitude within *A. californica*'s range. Therefore it is possible that heterogeneity in aridity along California's coast has influenced the population response to herbivore pressure, generating genetically based latitudinal clines in plant resistance.

Contributing to a scarce but growing body of literature, we report evidence for genetically based latitudinal clines in plant tolerance to herbivory. To our knowledge, there are only four within‐species studies to date that have quantified tolerance within a common garden comprised of populations sourced along a latitudinal gradient (Croy et al., 2020; Lehndal & Ågren, 2015; Sakata et al., 2017; Więski & Pennings, 2014). Two of these studies found no latitudinal relationship (Sakata et al., 2017; Więski & Pennings, 2014) and the other two found positive relationships between latitude and tolerance (Croy et al., 2020; Lehndal & Ågren, 2015). Specifically, Croy et al. (2020) found that tolerance exhibited a U‐shaped relationship with latitude, illustrating that tolerance can take on nonlinear relationships along environmental gradients; more studies that investigate evolutionary drivers of spatial variation in tolerance are needed. Moreover, tolerance is often assumed to be positively correlated with plant growth rate (Coley et al., 1985), and, although sometimes growth is linked to tolerance (Gianoli & Salgado‐Luarte, 2017), fast growth strategies may incur costs to belowground storage or other traits related to increased tolerance (Rose et al., 2009; Strauss & Agrawal, 1999). We found contrasting relationships between tolerance and growth and resource availability. However, we only measured aboveground biomass, and it is therefore possible that northern populations invest more in belowground storage. Trade‐offs or null relationships between tolerance to herbivory and growth rate have been documented elsewhere (Croy et al., 2020; Turley et al., 2013), and therefore growth rate may not serve as a reliable estimate of plant tolerance.

Although the patterns of interpopulation variation in growth differed between the two common gardens, plants from low‐resource (southern) environments were, on average, faster‐growing than plants from high‐resource (northern) environments. Intraspecific variation in *A. californica* growth response to mesic conditions (i.e., G × E) has been demonstrated with respect to both an irrigation treatment (Pratt & Mooney, 2013) and over 8 years of interannual variation in precipitation (Croy et al., 2021). However, in contrast with past studies finding greater plastic increases in growth for southern versus northern populations, we found that, although southern plants grew more on average, they experienced a greater reduction in growth when growing in a mesic versus xeric common garden. One possible explanation for the differences in growth patterns is that environmental conditions across common gardens vary beyond differences in aridity solely (e.g., soil, arthropods), and these conditions in the northern common garden might impose greater stress to southern versus northern populations. We have also observed in a multicommon garden analysis that transplanting populations both northward and southward results in reduced plant size (J. R. Croy and K. A. Mooney, unpublished data), suggesting that growth is a product of adaptation to particular environmental conditions. Altogether, these observations indicate that general trends between plant growth and resource availability may be difficult to detect, given plant adaptation to local conditions.

### Resource availability and plant growth and defense

Resource availability has been proposed to influence the evolution of plant defense strategies through both a bottom‐up (Coley et al., 1985; Endara & Coley, 2011; Fine et al., 2004) and feedback (Baskett & Schemske, 2018; Hahn & Maron, 2016; Moreira et al., 2017; Pellissier et al., 2014) dynamic. In the bottom‐up dynamic, the increased selection for plant tolerance associated with increasing resource availability reduces the selection for plant resistance to herbivory, generating a trade‐off between tolerance and resistance. In contrast, a feedback dynamic is possible if resource availability increases plant productivity and, subsequently, herbivore pressure, leading to increased selection on resistance and the co‐expression of plant tolerance and resistance. However, positive tolerance–resistance patterns might arise due to molecular constraints on tolerance–resistance trade‐offs, given that both have been shown to share the same molecular pathway (Mesa et al., 2017, 2019; Scholes & Paige, 2015). For example, Mesa et al. (2017) demonstrated that chromosomal plasticity (i.e., endoreduplication) varied across Arabidopsis genotypes and the degree of endoreduplication was linked positively to both plant tolerance and chemical resistance. Therefore, plant evolutionary response to the herbivores along a resource gradient might be molecularly constrained.

Our assessment of vertebrate herbivore pressure found that herbivore pressure did not vary with latitude, although the trend was positive. Similarly, past work on this system suggests that insect herbivory increases monotonically with latitude (higher in the north) (Pratt et al., 2017). The fact that northern populations from high‐resource environments have both lower resistance and higher (insect) herbivore densities suggests that herbivore populations respond to variation in plant quality, but this variation in herbivore pressure does not in turn feedback to alter selection for resistance. We speculate that greater resource availability of northern populations results in selection for increased tolerance, and this in turn decreases the selection for resistance with bottom‐up consequences for associated insect communities.

Although our study provides support for a strictly bottom‐up effect of resource availability on plant tolerance and resistance, there is growing empirical support for resource‐driven, top‐down effects of herbivory driving plant growth and defense patterns (Buckley et al., 2019; Hahn et al., 2018; Hahn & Maron, 2016; Kergunteuil et al., 2019; López‐Goldar et al., 2020; Pellissier et al., 2012, 2014). These two dynamics ultimately differ in their assumption about herbivore pressure across resource environments, with the former assuming inconsequential variability in herbivory (Coley et al., 1985) and the latter assuming that favorable environmental conditions augment herbivore pressure (Coley & Aide, 1991; Hahn & Maron, 2016; Janzen, 1970; Moreira et al., 2017). Hahn and Maron (2016) note that the former and latter pathways have been primarily explored in among‐ versus within‐species contexts and that the methodological approaches in which each pathway has been studied differ as well. Among‐species tests generally compare plants growing in adjacent high‐ versus low‐resource environments (e.g., light vs. gap specialists), whereas within‐species tests are generally performed along broad environmental gradients (e.g., elevational gradients). The methodological differences between among‐ versus within‐species studies suggest that the pathway through which resource availability influences plant growth and defense may not depend on taxonomic scale at all. For instance, although resource availability might influence herbivore pressure at small spatial scales, herbivore movement among patches (i.e., herbivore spillover) probably dilutes differences in herbivore pressure. In contrast, resource availability over larger spatial scales might limit herbivore spillover across plant populations and species, leading to gradients in both resource availability and herbivore pressure. Therefore, it is possible that dynamics shift from strictly bottom‐up effects toward feedback effects as the resource gradient and spatial scale increase in magnitude. Overall, more studies examining intraspecific and interspecific variation in plant growth, tolerance, and defense over broad environmental gradients are needed to uncover the evolutionary effects of resource availability on plant growth‐defense strategies.

### Concluding remarks

Altogether, our findings provide support for a latitudinal resource gradient shaping genetically based variation in plant growth and multivariate defense strategies among populations of a foundational shrub species. Given the evidence reported here and elsewhere for local adaptation to aridity in this system (Pratt & Mooney, 2013; Pratt et al., 2014; Croy et al., 2021; J. R. Croy and K. A. Mooney, unpublished data), climate change‐induced shifts in the aridity landscape are likely to lead to corresponding shifts in the defensive character of local *A. californica* populations. Populations from low‐resource environments have been shown to support lower densities and different communities of arthropods (Croy et al., 2021; Pratt et al., 2017), and therefore entire ecological communities might be altered as populations adapt to a changing climate. By linking environmental conditions to genetic variation in organismal traits that mediate ecological interactions, we can gain better insight into the evolutionary consequences of climate change.

## CONFLICT OF INTEREST

The authors declare no conflict of interest.

## Supporting information


Appendix S1

Appendix S2

Appendix S3

## Data Availability

Data and code (Croy et al., 2022) are available on Zenodo at https://doi.org/10.5281/zenodo.6522256.
