# Supplementary material for: Latitudinal resource gradient shapes multivariate defense strategies in a long‐lived shrub
Source: Ecology. 2022 Sep 29;103(12):e3830. doi: 10.1002/ecy.3830 (PMC10078560; doi:10.1002/ecy.3830)
Supplement: Supplementary file 1 — Appendix S1 [file ECY-103-0-s003.pdf]

Jordan R. Croy, Jessica D. Pratt, and Kailen A. Mooney

Latitudinal resource gradient shapes multivariate defense strategies in a long-lived shrub

Ecology

Appendix S1: Distribution of *Artemisia californica* populations used in this common-garden study, details on common garden design, and additional details on the methods for quantifying latitudinal variation in climate, soil, and herbivore pressure.

Table S1. Population locations and climate data (mean annual temperature and precipitation) for the 21 populations of *Artemisia californica* used in this study. The 21-population and 5-population gardens were established in 2011 and 2009, respectively.

Population Location		*Local Climate*		*Gardens*	*Sample Size*
Latitude (°)	Longitude (°)	MAT (°C)	MAP (cm)		
32.87	-117.25	16.9	26.6	2009, 2011	7, 10
33.05	-117.29	16.1	27.2	2011	9
33.65	-117.89	17.6	29.9	2011*	10
34.04	-118.6	16.9	42.1	2009, 2011	20, 10
34.06	-118.99	17.1	41.0	2011	10
34.32	-119.39	16.1	49.5	2011	10
34.41	-119.85	15.1	46.1	2011	10
34.46	-120.02	14.8	48.3	2011	11
34.5	-120.5	13.8	37.9	2011	10
34.68	-120.6	13.3	33.6	2011	10
35.15	-120.65	14.7	44.2	2011	10
35.44	-120.89	14.1	44.3	2009, 2011	15, 10
35.65	-121.23	13.4	54.1	2011	10
35.9	-121.46	14.3	73.6	2011	10
36.29	-121.84	14.1	91.6	2011	10
36.97	-122.12	12.1	69.3	2009, 2011	15, 10
37.3	-122.4	11.9	68.9	2011	10
37.59	-122.51	13.4	66.3	2011	10
37.83	-122.54	13.4	69.8	2009, 2011	14, 10
37.86	-122.57	13.7	84.7	2011	9
38.25	-122.96	12.7	90.1	2011	9
38.45	-123.12	11.6	107.6	2011	9

* *Location of southern common garden and* A. californica *population*
†*Location of northern common garden only (no* A. californica *population from this site)*

Table S2. Description of the data collected within each common garden.

Common garden	*Location*	*# Populations*	*Measurements made*
2009 Garden	Newport Beach, CA	5	• visual estimates of vertebrate herbivory
2011 Garden (South)	Newport Beach, CA	21	• plant damage surveys<br>• tolerance experiment<br>• growth<br>• leaf traits
2011 Garden (North)	Jenner, CA	21	• growth

Section S1. Common garden construction

The common garden established in 2009 (hereafter the "2009 garden") is also described in detail elsewhere (Pratt and Mooney 2013, Pratt et al. 2014, 2017). In spring 2008, we collected 20 cuttings from 20 *A. californica* plants in each of five source populations distributed along a coastal gradient. To minimize non-genetic maternal effects associated with plants cloned from cuttings (Roach and Wulff 1987), rooted cuttings were grown in the greenhouse and common garden for a total of 24 months before collecting data. In December 2009, the common garden was planted into three blocks containing a pair of plots, one irrigated and the other unirrigated (Pratt and Mooney 2013, Pratt et al. 2014, 2017). This study focuses primarily on the unirrigated plots The plants from each source population (sample sizes ranging from 7 to 21 per population) were evenly distributed among plots and randomized within each plot.

The common garden established in 2011 (hereafter the "2011 garden") is immediately adjacent to the 2009 garden. In December 2010, we collected seed from 10 *A. californica* plants in each of 21 source populations, including the five populations sampled for the 2009 garden. Seeds were germinated in early February 2010 in a greenhouse. In February 2011, when the plants reached a canopy volume of approximately 1,000 cm^3 (≈ 10 x 10 x 10 cm), they were transplanted to the common garden site with approximately ten individuals per population (N = 210 plants total), each from a unique seed mother. Plants were randomly assigned to locations within a 14 by 15 m grid, with each plant separated by 1.0 m from its closest neighbor. Plants within each garden were lightly irrigated during their first summer following transplant to increase survival.

Section S2. Methods for calculating aridity across sites.

We extracted PRISM climate data from 1970-2000 (4km spatial resolution). These climate variables were then used to calculate potential evapotranspiration (PET) using the Hargreaves equation per the protocol described by the Consortium for Spatial Information (CGIARCSI) Global Aridity and PET database (https://cgiarcsi.community/2019/01/24/global-aridity-index-and-potential-evapotranspiration-climate-database-v2/). The following equation was used to calculate monthly PET:

$$PET = 0.0023 * RA * (Tmean + 17.8) * TD^{0.5}$$

where RA is extraterrestrial radiation (on top of atmosphere), which was extracted from the R package sirad (Bojanowski 2016). RA was then converted from MJ/m-squared per day to mm/day by multiplying by 0.408 (Allen et al. 1998). As with the CGIARCSI (which is based on, Allen *et al.* 1998), we extracted RA on the 15th day of each month for each population, and then multiplied by the number of days in each month to achieve the mm/month equivalent. Tmean is monthly average temperature for each population. TD is the monthly temperature range (maximum – minimum). The unitless Aridity Index (mean annual precipitation/ mean annual potential evapotranspiration) is the inverse of aridity, with low values indicating more arid locations.

Table S3. List of nine soil properties extracted from the USDA NRCS SSURGO database, the variable name as listed in the "chorizon" table, and the descriptions provided by the USDA.

Abbreviation	*SSURGO Variable Name*	*Variable description*†
Sand	sandtotal_r‡	Mineral particles 0.05mm to 2.0mm in equivalent diameter as a weight percentage of the less than 2 mm fraction
Silt	silttotal_r	Mineral particles 0.002 to 0.05mm in equivalent diameter as a weight percentage of the less than 2.0mm fraction
Clay	claytotal_r	Mineral particles less than 0.002mm in equivalent diameter as a weight percentage of the less than 2.0mm fraction
OM	om_r	The amount by weight of decomposed plant and animal residue expressed as a weight percentage of the less than 2 mm soil material
Ksat	ksat_r	The amount of water that would move vertically through a unit area of saturated soil in unit time under unit hydraulic gradient
K	kffact	An erodibility factor which quantifies the susceptibility of soil particles to detachment by water
CEC	cec7_r	The amount of readily exchangeable cations that can be electrically adsorbed to negative charges in the soil, soil constituent, or other material, at pH 7.0, as estimated by the ammonium acetate method
pH	ph01mcacl2_r	The negative logarithm to base of 10 or the hydrogen ion activity in the soil, using the 0.01M CaCl2 method, in a 1:2 soil:solution ratio. A numerical expression of the relative acidity or alkalinity of a soil sample. (SSM)
AWC	awc_r	The amount of water that an increment of soil depth, inclusive of fragments, can store that is available to plants. AWC is expressed as a volume fraction, and is commonly estimated as the difference between the water contents at 1/10 or 1/3 bar (field capacity) and 15 bars (permanent wilting point) tension and adjusted for salinity, and fragments.

† Variable descriptions from the "chorizon" table within the USDA NRCS SSURGO database (Soil Survey Staff n.d.)

‡ r indicates that the values extracted were representative for the horizon.

Section S2. Description of the methods for extracting soil characteristics from each *Artemisia californica* site.

Methods

Using the USDA NRCS SSURGO database, we extracted nine physical attributes thought to be relevant for plant performance. These attributes are listed in Table S2. Each population occurs within a distinct soil type called a map unit. Each map unit is comprised of various soil components (component units), and the proportion of each component unit varies depending on the map unit. Moreover, each component unit contains unique soil horizon data. Soil properties were specifically extracted from the "chorizons" table within the SSURGO database. The chorizons table contains information on soil attributes at various soil depths, but because the majority of *A. californica* roots are concentrated within the first 50 cm of soil (Goldstein and Suding 2014), we computed weighted means for each of the nine attributes by soil depth. For instance, if the first soil layer depth was 30 cm and the second 20 cm, the attributes would be weighted accordingly. Next, to account for the varying amounts of component units within a map unit, we computed another weighted mean of the nine attributes weighted by component unit percentage. We were able to extract soil properties for 17 sites, and these results are plotted in Figure S2.

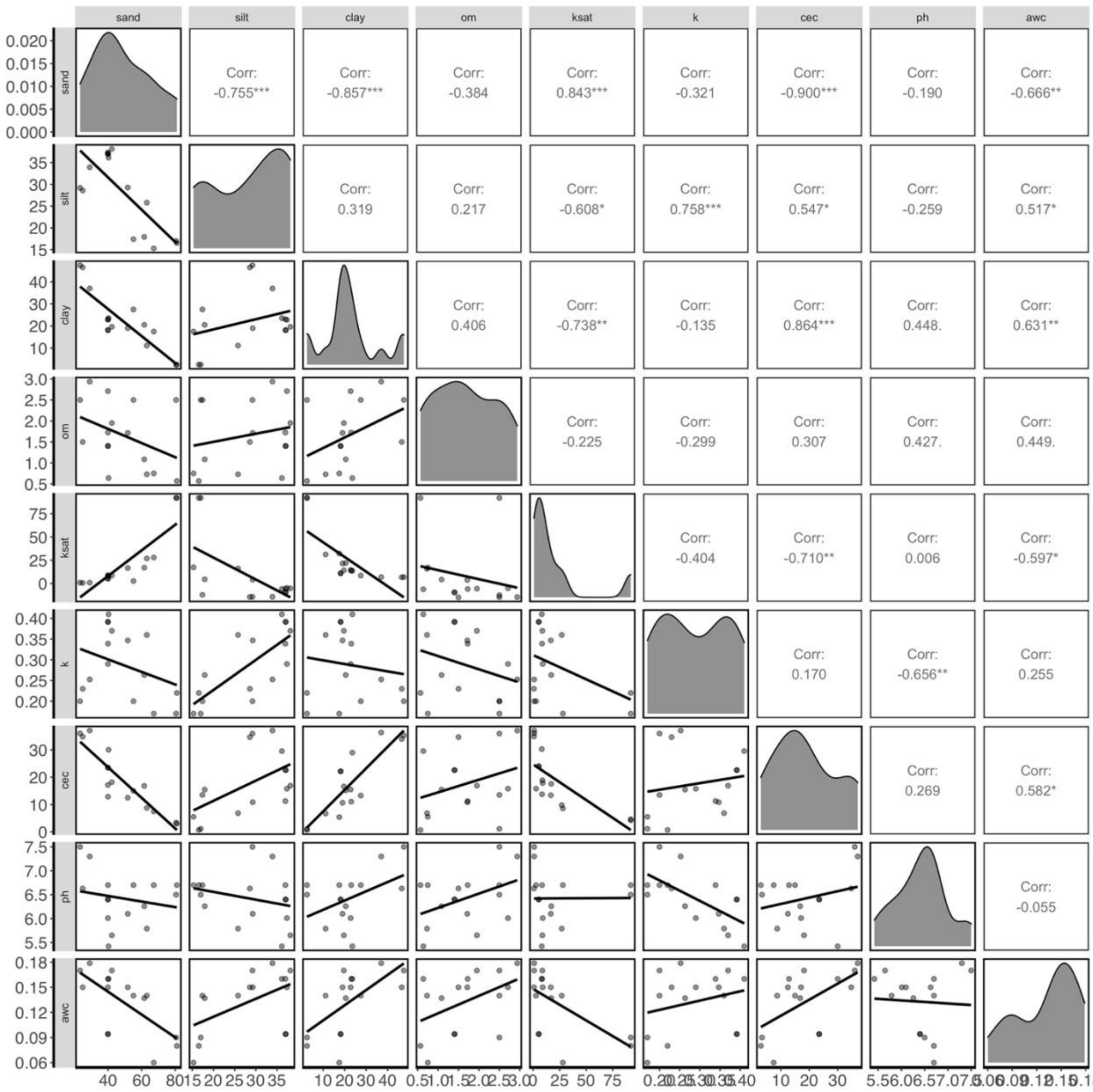

Figure S2. Pairwise relationships for the nine soil properties extracted from the USDA Soil SSURGO database from each *Artemisia californica* site used in this study. Histograms occupy the diagonal panels, correlation coefficients and their significance occupy the upper panels, and scatterplots for each pairwise relationship are shown in the lower panels.

Section S3. Description of the methods for estimating vertebrate herbivore pressure for each *Artemisia californica* site.

To estimate herbivore pressure along the coast, we extracted iNaturalist (*iNaturalist*) observations within a 5 km^2 square around each population source location along the California coast using the rinat package in R (Barve et al. 2021). Because the vertebrate herbivores that feed on *Artemisia californica* are herbivorous mammalian taxa, we collected the total number of mammalian observations and filtered them by known *A. californica* herbivores (Litle et al. 2019) and potential *A. californica* herbivores (Appendix S1: Table S2). To account for substantial variation in sampling effort along the coast, we also collected the total number of observations of reptiles, birds, and insects. The total number of vertebrate herbivore observations were divided by the sum of total reptile, bird, insect, and non-herbivore mammal observations to account for variation in sampling effort across sites. Variation in arthropod herbivore pressure along the coast has previously been presented for five wild-sampled *A. californica* populations distributed along a latitudinal gradient (Pratt et al. (2017).

Table S4. List of herbivorous mammalian taxa known (or likely) to feed on *Artemisia californica* that were extracted from iNaturalist to estimate herbivore pressure across *A. californica* populations.

Vertebrate herbivore taxa

Artiodactyla	*Dipodomys stephensi*	*Neotoma bryanti*	*Peromyscus*
Arvicolinae	*Dipodomys venustus*	*Neotoma fuscipes*	*Peromyscus californicus*
Bovidae	*Geomyidae*	*Neotoma fuscipes annectens*	*Peromyscus fraterculus*
Capra hircus	*Heteromyidae*	*Neotoma lepida*	*Peromyscus leucopus*
Capreolinae	*Lagomorpha*	*Neotoma macrotis*	*Peromyscus maniculatus*
Caprini	*Leporidae*	*Neotominae*	*Peromyscus maniculatus anacapae*
Cervidae	*Lepus*	*Neotomini*	*Rattus*
Cervus	*Lepus americanus*	*Ochotona princeps*	*Rattus norvegicus*
Cervus canadensis	*Lepus californicus*	*Odocoileus*	*Rattus rattus*
Cervus canadensis canadensis	*Marmota monax*	*Odocoileus hemionus*	*Reithrodontomyini*
Cervus canadensis nannodes	*Marmotini*	*Odocoileus hemionus californicus*	*Reithrodontomys*
Cervus nippon	*Microtini*	*Odocoileus hemionus columbianus*	*Reithrodontomys megalotis*
Chaetodipus	*Microtus*	*Odocoileus hemionus fuliginatus*	*Ruminantia*
Chaetodipus californicus	*Microtus californicus*	*Odocoileus virginianus*	*Sciurus niger*
Chaetodipus fallax	*Microtus californicus californicus*	*Oryctolagus*	*Sciurus niger rufiventer*
Cricetidae	*Microtus pennsylvanicus*	*Oryctolagus cuniculus*	*Sigmodon hispidus*
Didelphimorphia	*Muridae*	*Oryctolagus cuniculus domesticus*	*Sus scrofa*
Dipodomyinae	*Murinae*	*Otospermophilus*	*Sylvilagus*
Dipodomys	*Muroidea*	*Otospermophilus beecheyi*	*Sylvilagus audubonii*
Dipodomys agilis	*Mus*	*Ovis*	*Sylvilagus bachmani*
Dipodomys heermanni	*Mus musculus*	*Ovis aries*	*Sylvilagus bachmani cinerascens*
Dipodomys merriami	*Myomorpha*	*Perognathinae*	*Thomomys*
Dipodomys simulans	*Neotoma*	*Perognathus*	*Thomomys bottae*

Figure S3. Latitudinal clines in estimated vertebrate herbivore pressure using *iNaturalist* observations within a 5 km^2 square around each site.

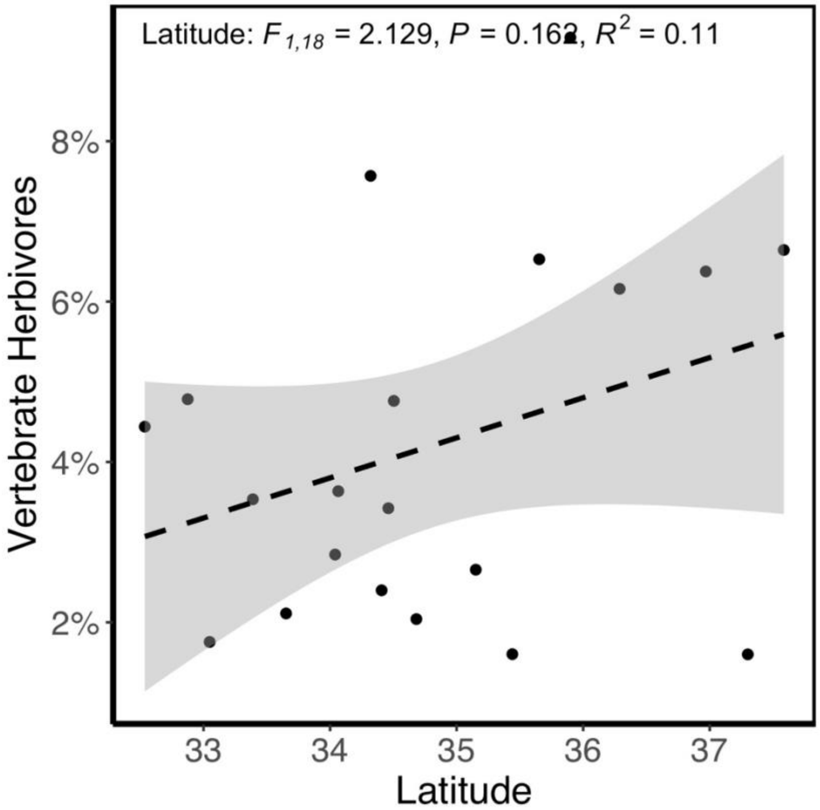

Literature Cited

Allen, R. G., L. S. Pereira, D. Raes, and M. Smith. 1998. Crop evapotranspiration: Guidelines for computing crop requirements. FAO, Rome, Italy.

Barve, V., E. Hart, and S. Guillou. 2021. rinat: Access “iNaturalist” Data Through APIs.

Bojanowski, J. S. 2016. sirad: Functions for Calculating Daily Solar Radiation and Evapotranspiration.

Goldstein, L. J., and K. N. Suding. 2014. Applying competition theory to invasion: resource impacts indicate invasion mechanisms in California shrublands. Biological Invasions 16:191–203.

iNaturalist. Available from https://www.inaturalist.org. Accessed [Mar 2021]. (n.d.). .

Litle, J., L. H. Quon, M. L. Antill, E. J. Questad, and W. M. Meyer. 2019. Vertebrate herbivory on shrub seedlings in California sage scrub: important but understudied interactions. Plant Ecology 220:523–528.

Pratt, J. D., A. Datu, T. Tran, D. C. Sheng, and K. A. Mooney. 2017. Genetically based latitudinal clines in Artemisia californica drive parallel clines in arthropod communities. Ecology 98:79–91.

Pratt, J. D., K. Keefover-Ring, L. Y. Liu, and K. A. Mooney. 2014. Genetically based latitudinal variation in Artemisia californica secondary chemistry. Oikos 123:953–963.

Pratt, J. D., and K. A. Mooney. 2013. Clinal adaptation and adaptive plasticity in Artemisia californica: implications for the response of a foundation species to predicted climate change. Global Change Biology 19:2454–2466.

Roach, D. A., and R. D. Wulff. 1987. Maternal Effects in Plants. Annual Review of Ecology and Systematics 18:209–235.

Soil Survey Staff. (n.d.). Soil Survey Geographic (SSURGO) Database. https://sdmdataaccess.sc.egov.usda.gov/?referrer=Citation.htm-SSURGOLink.
