# Supplementary material for: Latitudinal resource gradient shapes multivariate defense strategies in a long‐lived shrub
Source: Ecology. 2022 Sep 29;103(12):e3830. doi: 10.1002/ecy.3830 (PMC10078560; doi:10.1002/ecy.3830)
Supplement: Supplementary file 2 — Appendix S2 [file ECY-103-0-s002.pdf]

Jordan R. Croy, Jessica D. Pratt, and Kailen A. Mooney

Latitudinal resource gradient shapes multivariate defense strategies in a long-lived shrub

Ecology

Appendix S2. Details on the extraction, identification, and quantification of *Artemisia californica* leaf terpenes.

To assess terpene concentrations, we randomly collected at least 10 leaves and immediately placed them into 2 ml n-hexane, sonicated for 10 min and soaked at room temperature. After seven days, extracts were poured off and stored at -80 °C until analysis by gas chromatography and mass spectrometry (GC-MS) and leaf material was dried at 60 °C for 72 h and weighed. For terpene analysis, 10 µl of an internal standard solution (0.13 µl ml^{-1} m-xylene in n- hexane) was added to 90 µl of each sample extract. Samples were injected (4 µl) onto a GC-MS (Trace MS+, ThermoFisher Scientific, Asheville, NC) fitted with a 30 m x 0.25 mm x 0.25 µm film thickness DB-5 fused silica column (J&W Scientific, Folsom, CA). The GC was operated in splitless mode with helium as the carrier gas (flow rate 1 ml min^{-1}). The GC oven temperature program was as follows: 1 min hold at 50 °C, 5 °C min^{-1} ramp to 180 °C, 20 °C min^{-1} ramp to 290 °C, and 1 min hold at 290 °C. The mass spectrometer was operated in electron ionization mode and data were collected between m z^{-1} 50–650. We identified mono- and sesquiterpenes in our samples and examined relative investment in defense by calculating the normalized area per dry weight (peak area/area of internal standard/dry weight of leaf material) for all terpenoid compounds detected.
