# Supplementary material for: Latitudinal resource gradient shapes multivariate defense strategies in a long‐lived shrub
Source: Ecology. 2022 Sep 29;103(12):e3830. doi: 10.1002/ecy.3830 (PMC10078560; doi:10.1002/ecy.3830)
Supplement: Supplementary file 3 — Appendix S3 [file ECY-103-0-s001.pdf]

Jordan R. Croy, Jessica D. Pratt, and Kailen A. Mooney

Latitudinal resource gradient shapes multivariate defense strategies in a long-lived shrub

Ecology

Appendix S3. Latitudinal clines in the herbivore damage and tolerance across *Artemisia californica* populations.

Table S1. Statistical results for *A. californica* damage by vertebrate herbivores, plant biomass, and survival. Bold indicates P < 0.05.

Dependent Variable	Statistical model	Latitude of Origin		Clipping Treatment		Lat*Clip		initial plant size	
		χ^2	*P*-value	χ^2	*P*-value	χ^2	*P*-value	χ^2	*P*-value
Proportion chewed (ln-transformed)	linear mixed effects	**7.52**	**0.006**	-	-	-	-	-	-
Total biomass of clippings (ln-transformed)	linear mixed effects	**6.77**	**0.009**	-	-	-	-	-	-
Plant growth rate	linear mixed effects	**5.81**	**0.016**	0.01	0.918	3.14	0.076	0.26	0.607
Plant survival	generalized linear mixed effects (link = binomial)	**10.12**	**0.002**	2.91	0.088	2.08	0.150	7.11	0.008

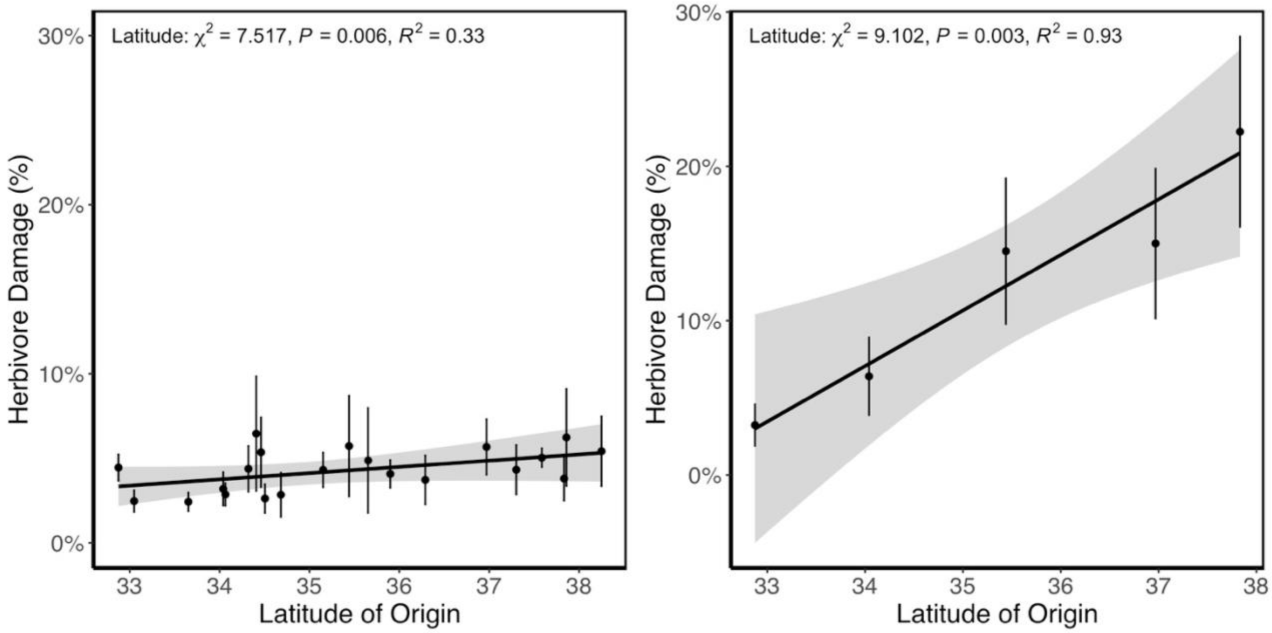

Figure S1. Accounting for variation in plant size, percent herbivory is plotted against plant latitude of origin for the two independent estimates. (Left) Percent herbivory was estimated in 2016 via clippings collected beneath *A. californica* canopies. (Right) Percent herbivory was estimated in 2010 via visual assessments of damage on individual plants. Least squares regression lines plotted with shaded regions indicating 95% C.I.

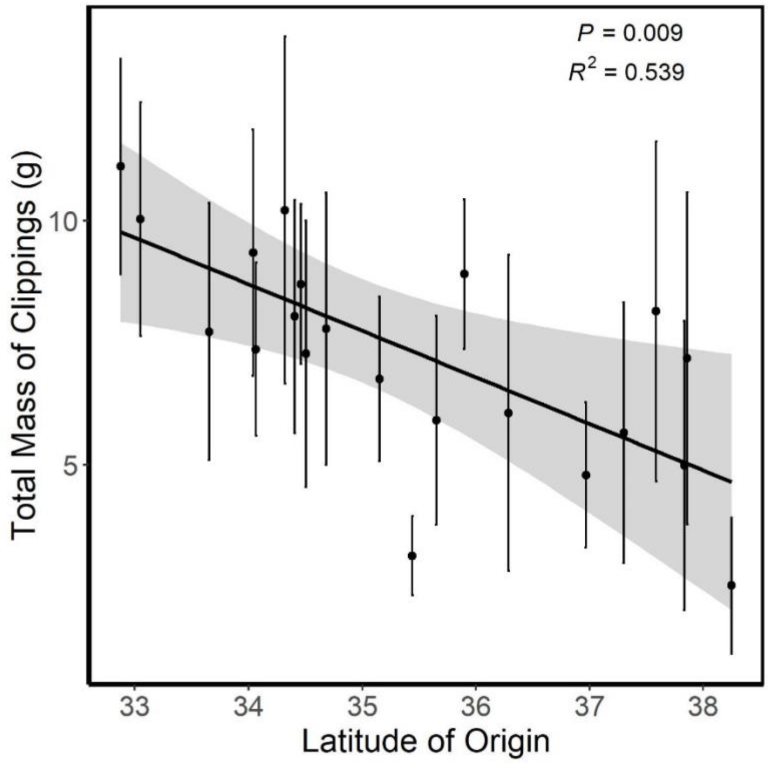

Figure S2. Latitudinal variation in the total mass of plant tissue removed by herbivores and collected from beneath a shrub (not accounting for plant size). Least squares regression lines plotted with shaded regions indicating 95% C.I.

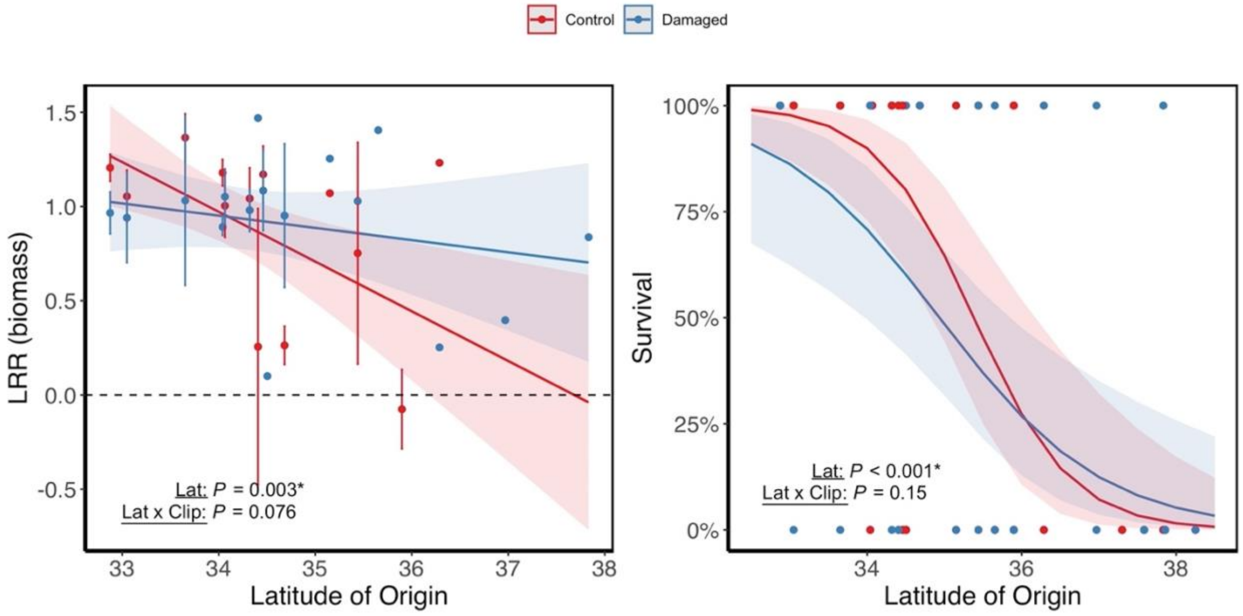

Figure S3. Latitudinal variation in *A. californica* growth rate (left) and survival (right) from 2016-2017 after the clipping treatment was implemented in June 2016. Growth was calculated as the log response ratio (LRR) of biomass in 2017 vs 2016, i.e., $\log(\left(\frac{biomass_{2016}}{biomass_{2017}}\right)$, where a horizontal line at zero indicates no growth occurred. A significant interaction between latitude and our clipping treatment would indicate genetic-based variation in tolerance associated with latitude Accordingly, we report p-values for the interaction.
